# Improved A* Algorithm for Mobile Robots under Rough Terrain Based on Ground Trafficability Model and Ground Ruggedness Model

**DOI:** 10.3390/s24154884

**Published:** 2024-07-27

**Authors:** Zhiguang Liu, Song Guo, Fei Yu, Jianhong Hao, Peng Zhang

**Affiliations:** 1 School of Control and Mechanical Engineering, Tianjin Chengjian University, Tianjin 300384, China; 2 School of Mechanical Engineering, Hebei University of Technology, Tianjin 300401, China; guosonghebut@163.com (S.G.); fyu@hebut.edu.cn (F.Y.); zhangpeng@hebut.edu.cn (P.Z.); 3 National Technological Innovation Method and Tool Engineering Research Center, Tianjin 300401, China; 4 Comprehensive Business Department, CATARC (Tianjin) Automotive Engineering Research Institute Co., Ltd., Tianjin 300300, China; haojianhong@catarc.ac.cn

**Keywords:** A* algorithm, path planning, elevation maps, rough terrain

## Abstract

Considering that the existing path planning algorithms for mobile robots under rugged terrain do not consider the ground flatness and the lack of optimality, which leads to the instability of the center of mass of the mobile robot, this paper proposes an improved A* algorithm for mobile robots under rugged terrain based on the ground accessibility model and the ground ruggedness model. Firstly, the ground accessibility and ruggedness models are established based on the elevation map, expressing the ground flatness. Secondly, the elevation cost function that can obtain the optimal path is designed based on the two types of models combined with the characteristics of the A* algorithm, and the continuous cost function is established by connecting with the original distance cost function, which avoids the center-of-mass instability caused by the non-optimal path. Finally, the effectiveness of the improved algorithm is verified by simulation and experiment. The results show that compared with the existing commonly used path planning algorithms under rugged terrain, the enhanced algorithm improves the smoothness of paths and the optimization degree of paths in the path planning process under rough terrain.

## 1. Introduction

In recent years, a comprehensive discipline with a focus on mobile robots has developed, focusing on the latest research on machinery, electronics, computers, automatic control, and artificial intelligence and other multidisciplinary research results, representing the highest achievement of mechatronics. Mobile robots usually exhibit high mobility performance, can replace humans in complex and dangerous environments, and thus often exhibit excellent maneuverability and flexibility compared to general robots [1]. Research on mobile robots has gradually changed from flat indoor environments to complex terrain environments, and mobile robots have been increasingly used in security and search and rescue [2], field operations [3], mineral exploration, deep-space exploration, and other environments [4]. Rough terrain refers to unstructured natural environments, which are different from indoor environments in that their ground characteristics are more complex and dangerous, and the terrain is rugged [5]. Therefore, mobile robots facing such environments are often equipped with complex adaptive mobility systems, which can effectively adapt to the ground and ensure the robot’s stable movement.

Scholars have conducted a lot of exploration and research on the path planning of mobile robots. Reference [6] presents a new global-best brainstorm optimization algorithm for constrained 3D UAV path planning, which can improve search capability and robustness. Zhong et al. presented a new methodology based on neural dynamics for optimal robot path planning that can work in real time and does not require prior knowledge [7]. Some scholars presented a heat conduction method for optimal robot path planning by drawing an analogy between mobile robots’ heat conduction and path planning. This method establishes an iterative heat flux method to generate an optimal path from the robot to the target [8].

Mobile robots that move over rough terrain have difficulty recognizing specific obstacles and feasible spaces due to the rugged terrain, thus posing challenges to the ground trafficability and stability of mobile robots. Therefore, in many cases, mobile robots that move on rough terrain often need a certain degree of autonomous decision-making and navigation capabilities to effectively plan and track an optimal, safe, and smooth path to reach the target point and complete designated tasks. Therefore, the path planning of mobile robots on rough terrain is an essential issue in mobile robot motion and navigation.

Some scholars have proposed corresponding improvement methods for the ground trafficability problem in mobile robot navigation under rough terrain. First, the rough terrain is modeled using machine learning or geometric features. Second, the corresponding cost function is established to quantify it. Finally, the optimal path is generated [9,10,11].

Based on the above method, the mobile robot can recognize the ground environment and realize smooth autonomous navigation to some extent. However, the above methods applied to mobile robots under rough terrain still need to solve the problem of center-of-mass stabilization. The center-of-mass instability of mobile robots under rough terrain is mainly caused by the following reasons: the ground model in the existing path planning algorithms must consider ruggedness and the lack of optimality of the algorithms built for the ground model. Therefore, designing an algorithm that ensures ruggedness and optimality is crucial to prevent the center-of-mass instability and achieve safe autonomous navigation.

In path planning algorithms, compared to other path planning algorithms, the A* algorithm is portable and exhibits plasticity, is simple and easy to implement, requires a small amount of computation, and has low complexity. Moreover, as long as starting and end points are defined along the path, the algorithm will undoubtedly find the optimal path. The A* algorithm is also more widely used in path planning and is suitable for algorithmic improvement [12]. Therefore, this paper will use the A* algorithm as the basic algorithm for improvement.

Based on the above, this paper proposes an improved A* algorithm for mobile robots under rough terrain based on the ground trafficability and ruggedness models. The algorithm establishes the ground trafficability model and ground ruggedness model based on elevation maps to be able to assess the ruggedness of the ground, thus ensuring that the mobile robot chooses the overall smooth ground and avoids the center-of-mass instability caused by the lack of smoothness of the ground model. After that, a continuous and specific cost function based on the ground trafficability model and the ruggedness model is designed to obtain the optimal path so that the mobile robot can avoid the center-of-mass instability caused by the non-optimal path.

The main novelties of this paper compared to the current algorithms are as follows:

1. A ground model that considers ground ruggedness is added to the ground model part, which improves the reflection of the ground environment and avoids the center-of-mass instability of the mobile robot due to the ground model’s failure to consider ground ruggedness, compared with the existing improved path planning algorithms based on the ground trafficability model.
2. A continuous cost function that conforms to the characteristics of the ground model is designed, which avoids the center-of-mass instability of the mobile robot due to the lack of optimality of the algorithm, compared with the path planning based on classification or priority calculation.

We provide a literature review of the related work in Section 2. Section 3 describes the specific improvement methodology, including the ground ruggedness model, the ground trafficability model, the cost function design based on these two models, and the particular flow of the improvement methodology. Section 4 contains the experimental results of the simulation environment and the analysis of the results. Section 5 contains the results of the physical experiments and the study of the experiments. Section 6 discusses the experimental results and analyzes the current problems. Section 7 summarizes the entire research and presents the outlook for future research.

## 2. Related Work

### 2.1. Path Planning Algorithms

Current path planning algorithms contain two main categories: the grid-based path planning method [13,14] and the sampling-based path planning method [15,16,17]. The grid-based path planning method is a method that builds a map based on ground information and assigns a specific cost to each grid so that the search is conducted step by step until the optimal path is obtained. The sampling-based method is mainly applicable to mobile robots with kinematic constraints. It can obtain a route that fully conforms to the kinematic constraints of the mobile robot. Still, based on the sampling-based method, it results in a local optimum, generating a non-optimal path. Therefore, the grid-based path planning method is often used for rough terrain mobile robots.

Several scholars have addressed the shortcomings of path planning for mobile robots under rough terrain, i.e., ground trafficability is not considered, and the evaluation of paths is only assigned according to cost using distance [18,19,20] which results in the inability to reflect the ruggedness degree of the ground. Therefore, improving the path planning algorithm for rough terrain mobile robots is mainly carried out by building a ground model that recognizes rough terrain, in addition to designing a cost function so that the ground model can quantify ground trafficability.

### 2.2. Ground Model

Before establishing the ground model, it is necessary to conduct environmental modeling. In the environmental modeling of the most widely used elevation maps, elevation maps are based on grid maps with elevation values instead of occupancy information stored in each grid, which does not occupy the grid maps [21]. Moreover, octree maps [22] can represent a 2.5-dimensional ground with grid maps, and they can also be used to ensure that the number of search nodes is acceptable to prevent slow searches. Therefore, elevation maps are widely used to navigate rugged and complex ground [23]. However, since the elevation map is only a 2.5-dimensional map, which can only display the elevation information of each piece of ground, existing algorithms for planning using elevation maps [24] are limited to using 3-dimensional distance to describe the degree of ground undulation; moreover, the algorithm cannot reflect the specific ground’s trafficability.

Concerning the low expression of the degree of ground undulation on elevation maps, many scholars have improved the design of ground models based on elevation maps to solve the shortcomings of the traditional global path planning algorithm. The improvement in the ground model based on elevation maps mainly consists of two methods: The first is using a ground model based on machine learning, which is obtained by training point cloud information. However, machine learning simplifies the design of the ground model of the rough terrain mobile robot, particularly the training model constructed using the deep reinforcement learning method, which is also a popular research direction. Safety measures and poor interpretability for non-specific ground are also prone to more significant problems; thus, the rough terrain machine learning-based ground model is applied less. Another method is using a ground model based on geometric features. The ground model’s design is mainly based on the characteristics of mobile robots and elevation maps, and it indicates the degree of smoothness of the route. This method is continuously improving applications and is a current research focus.

Ground modeling based on geometric features has been researched by several scholars, among which Chen Gang [4] modeled and quantified the passing difficulty of the ground based on elevation maps through point cloud information, considering factors such as the slope of the robot. Xiaowei L [25] proposed a slope and obstacle crossing model based on the establishment of elevation maps and further improved the passing difficulty of the ground based on robot dynamics. Dianhua Zhang [26] proposed a slope, roughness, and elevation difference construction model based on elevation maps to refine the passing difficulty of the ground further. Hines T [27] proposed a virtual surface based on elevation maps and the sensor characteristics of a mobile robot, thus further solving the unclear recognition of negative obstacle navigation. Although the above improvements to the ground model further improve the mobile robot’s recognition of ground trafficability, some things can still be improved. The above algorithm for enhancing the ground model based on elevation maps only constructs the ground model for the point cloud information within a single grid. Still, it does not consider the smoothness of the mobile robot and only creates a ground model for a single grid.

### 2.3. Cost of the Ground Model

In addition to improving the ground model based on elevation maps, further cost designs of the ground model are needed. The current cost design methods based on the ground model mainly include the cost based on machine learning [28,29], the cost based on fuzzy logic or precedence comparisons [25,30,31], and the cost function of the potential field based on the obstacle’s establishment [32]. Although the cost based on machine learning has a specific continuous cost, it requires a priori data acquisition and, therefore, advanced experiments. Thus, it does not apply to rough terrain mobile robots. For the cost based on fuzzy logic or priority comparisons, although its computation is faster, a ground model that differs significantly in results and is equal to the exact cost is not the optimal path. The cost function of the potential field established based on obstacles [12,17] could be more optimal. The cost function of the potential field selected based on obstacles is currently used more, but the degree of ground undulation compared to the possible field of this cost needs to be more consistent with the characteristics of layer-by-layer reduction. Simultaneously, it cannot show that the ground’s trafficability can be overcome. Moreover, the specific ground undulation ultimately cannot be obtained.

Therefore, the current improved path planning algorithm based on the ground model has a low optimality problem. This results in selecting paths with low ground trafficability, leading to the mobile robot’s center-of-mass instability.

## 3. Improved A* Algorithm

**Figure 1:**
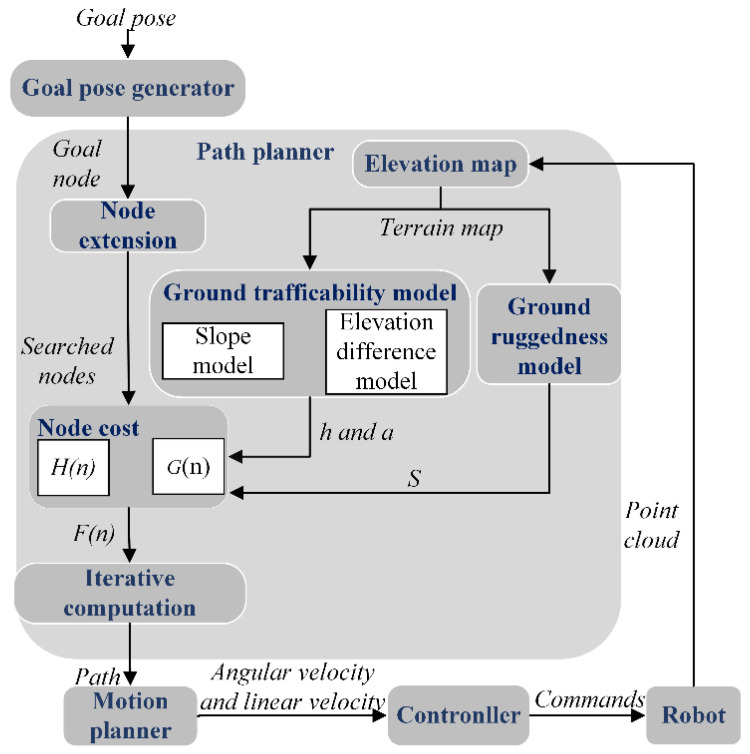
The autonomous navigation system framework. The mobile robot establishes an elevation map and a target node based on the goal pose and point cloud information, after which a new path is generated by the path planner module and tracked by the controller until the mobile robot reaches the node where the goal pose is located.

According to the traditional navigation system and the characteristics of the improved algorithm in this article, the navigation system framework constructed in this article is shown in Figure 1. First, the navigation system sends a target attitude to the target attitude generator and then sends the target node through the target attitude generator to drive the path planner module. The navigation system then creates an elevation map of the mobile robot based on the point cloud information obtained from the 3D LiDAR of the mobile robot. Then, the child nodes are received by an eight-directional search based on the coordinates of the target node and the coordinates of the starting node of the mobile robot. 

The ground trafficability model and ground ruggedness model created by the elevation map are used to calculate each node, and the slope *a*, height difference *h*, and ground ruggedness *S* of each node are obtained. Then, the path cost is calculated, and the cost functions *G*(*n*) and *H*(*n*) are calculated based on *a*, *h*, *S*, and the path distance, and the cost *F*(*n*) is obtained by weighted calculation. The node with the lowest cost is selected from these eight sub-nodes until the search reaches the goal point, and finally, the path with the lowest cost is output. Ultimately, it is also necessary to output the path to the mobile robot to drive it to move. Still, the path output from the path planning module cannot be directly applied to the mobile robot, so it needs further processing to output the optimal path as velocity information through the motion planner and output the expected velocity information to the underlying controller of the mobile robot to drive the mobile robot to move to the specified position.

### 3.1. Ground Trafficability Model

The ground trafficability model refers to a model that describes how difficult it is for mobile robots to pass through a path formed between a parent node and a child node. Environments that are difficult for mobile robots to pass through include sloping ground under rough terrain, separate protruding land under rough terrain, and depressed ground, including steps, equipment, and potholes. Therefore, to address these characteristics, elevation maps will be utilized to model height differences and slopes to determine if the search ground is passable and how difficult it is to pass through.

**Figure 2:**
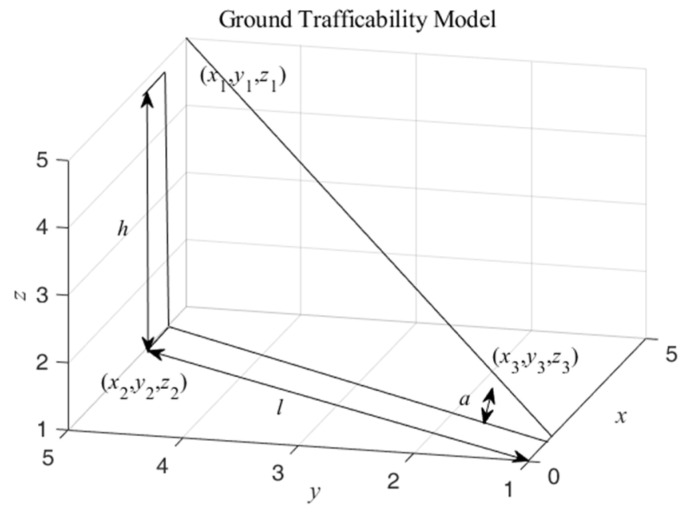
Ground trafficability model. The elevation difference model represents the difference between the child and parent nodes, where (*x*_1_, *y*_1_, *z*_1_) and (*x*_2_, *y*_2_, *z*_2_) represent the parent and child node coordinates, respectively. The slope model represents the slope between the child node and the parent node, and (*x*_1_, *y*_1_, *z*_1_) and (*x*_3_, *y*_3_, *z*_3_) represent the parent and child node coordinates, respectively.

The elevation difference model is the elevation difference between the parent node and the child node, and the difficulty of passing is judged according to the characteristics of the mobile robot. Since the coordinates of the child node are (*x_i_*, *y_j_*, *z_i_*_,*j*_), it is necessary to judge the z-difference between the coordinates of the child node and the coordinates of the parent node to obtain the elevation difference *h* to consider whether it is possible to pass and the difficulty of passing. According to this feature, assuming that the parent node coordinates are (*x*_1_, *y*_1_, *z*_1_) and the child node coordinates are (*x*_2_, *y*_2_, *z*_2_), the corresponding elevation difference model is established, as shown in Figure 2. 

Then, the related *h* is obtained based on these data, as shown in (1).

(1) $$h=\left|z_{1}-z_{2}\right|$$

In contrast to the elevation difference model, the slope model refers to the inclination angle formed by the parent and child nodes. Then, the difficulty of passage is determined based on the attributes of the mobile robot and the characteristics of the rough terrain. Similarly, since the center coordinates of each node in the elevation map are (*x_i_*, *y_j_*, *z_i_*_,*j*_), it is necessary to determine the difference in *z*-values between the parent node coordinates and the coordinates of the child nodes, as well as the distance *l* in the xoy plane, to obtain the corresponding tangent values, and thus the slope *a*, to determine the difficulty of passage.

Afterward, according to this characteristic and the above assumption of the elevation difference model to establish the corresponding slope model, as shown in Figure 2, according to the above (1), we obtain the *h* of the existing elevation difference model. After that, according to the coordinates of the two points (*x*_1_, *y*_1_, *z*_2_) and (*x*_2_, *y*_2_, *z*_2_), we obtain the distance between the two points. Finally, the specific formula for the slope *a* is shown in (2).

(2) $$a=arctan\frac{h}{\sqrt{{\left(x_{1}-x_{2}\right)}^{2}+{\left(y_{1}-y_{2}\right)}^{2}}}$$

### 3.2. Ground Ruggedness Model

The ground ruggedness model refers to a model that describes the degree of fluctuation of the search ground. A mobile robot needs a specific running channel during its traveling process because a mobile robot is not a mass moving. Therefore, under rough terrain, if a mobile robot needs to travel smoothly, it is necessary to ensure the accessibility of the traveling area and to search for a local area with a low degree of ground undulation.

**Figure 3:**
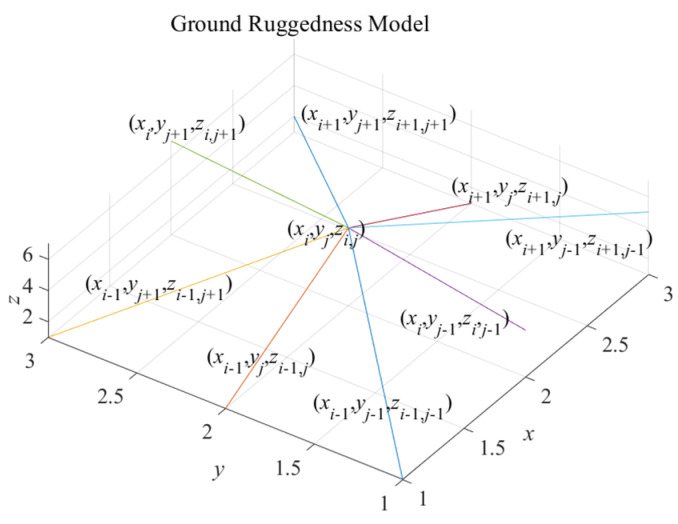
Ground ruggedness model. The ground ruggedness model describes the degree of fluctuation of the search ground. The child node is (*x_i_*, *y_j_*, *z_i_*_,*j*_).

Therefore, in this paper, a ground ruggedness model reflecting the degree of ground undulation is designed based on the above characteristics and the degree of terrain undulation. The ground ruggedness model will calculate the average elevation of the child nodes and the eight neighboring nodes of the child nodes and then calculate the variance in the elevation of the nine nodes based on the average elevation to represent the degree of ground undulation. Assuming that the coordinates of the child node are (*x_i_*, *y_j_*, *z_i_*_,*j*_), the corresponding ground ruggedness model is established, as shown in Figure 3.

Therefore, the following formula is established to calculate the search grid’s ground ruggedness: first, the *z*-axis coordinates of the surrounding grid centers will be summed to obtain the mean value *z_j_*, as shown in (3), and then the variance *S* will be received according to the variance formula, as shown in (4). The parameter *S* is the roughness of the ground.

(3) $$z_{j}=\frac{\left(\sum_{n=j-1}^{j+1}\sum_{m=i-1}^{i+1}z_{m,n}\right)}{9}$$

(4) $$S=\sum_{n=j-1}^{j+1}\sum_{m=i-1}^{i+1}(z_{m,n}-z_{j}{)}^{2}$$

### 3.3. Cost Function

Firstly, the path cost in the original A* algorithm does not consider the effect of ground accessibility and ground ruggedness on the path, so it is necessary to design a cost function that combines distance, ground accessibility, and ground ruggedness to show the effect on the path. Secondly, the existing elevation cost function uses a fuzzy logic function and a classification function, so the generated paths may not be optimal. Finally, *F*(*n*) in the A* algorithm consists of *G*(*n*) and *H*(*n*), and *H*(*n*) is a heuristic function mainly used to improve the search efficiency, so the improvement in *G*(*n*) will reflect the ground accessibility, ground ruggedness, and distance of the path. 

Therefore, it is necessary to design an elevation cost function to represent the degree of influence of the search grid ground on the path in *G*(*n*). Firstly, the elevation function needs a specific continuous cost to improve the algorithm’s optimality. Secondly, the elevation cost function needs to consider the characteristics of ground trafficability and ground ruggedness. Exceeding this threshold can mean that the mobile robot cannot pass. Thirdly, for the mobile robot, the slight fluctuation of the ground and the gently sloping steps are feasible and have little effect on the smooth movement of the mobile robot. Still, the closer it gets to the threshold value, the faster the difficulty of movement becomes until it is impossible to travel. Hence, the difficulty of ground travel and the inaccurate fluctuation grow faster and faster with the value increase.

(5) $$g(n)=\omega_{s}f_{s}+\omega_{e}f_{e}+\omega_{r}f_{r}$$

Therefore, the growth rate of the cost change will also change too fast. For the above characteristics and to improve the cost function’s calculation rate, the primary function’s tangent function is used as a prototype to improve the design of the elevation cost function *g*(*n*) corresponding to each node n. The elevation cost *g*(*n*) consists of the slope cost function *f*_s_, the elevation difference cost function *f*_e_, and the ruggedness cost function *f*_r_, and the corresponding weights are *ω*_s_, *ω*_e_, and *ω*_r_, where *ω*_s_, *ω*_e_, *ω*_r_, and 1. The formula for *g*(*n*) is shown in (5).

(6) $$f_{s}=\left\{\begin{array}{c} \tan\frac{\pi \times \left(a_{y}-a\right)}{2\times a_{y}}\left(a<a_{y}\right)\\ +\infty \left(a\geq a_{y}\right) \end{array}\right.$$

(7) $$f_{e}=\left\{\begin{array}{c} \tan\frac{\pi \times \left(h_{y}-h\right)}{2\times h_{y}}\left(h<h_{y}\right)\\ +\infty \left(h\geq h_{y}\right) \end{array}\right.$$

(8) $$f_{r}=\left\{\begin{array}{c} \tan\frac{\pi \times \left(S_{y}-S\right)}{2\times S_{y}}\left(S<S_{y}\right)\\ +\infty \left(S\geq S_{y}\right) \end{array}\right.$$

The slope cost function and elevation difference cost function are shown in (6) and (7), where *h* and *a* are the elevation value and slope from the child node (*x_i_*, *y_j_*, *z_i_*_,*j*_) to the parent node and *h_y_* and *a_y_* are the thresholds for the elevation difference and the slope of the mobile robot. Due to the different sampling methods of the model, the ruggedness cost function is shown in (9), where *S* is the ground ruggedness at the center of the child node, and *S_y_* is the threshold value of the ground ruggedness for the mobile robot to be able to ensure normal task travel. The final improved *G*(*n*) is shown in (9). In (9), *ω*_1_ and *ω*_2_ are the weights of the elevation cost and distance cost in the cost between the child node and the parent node, respectively, and *d*(*n*) represents the distance cost between the child node and the parent node.

(9) $$G\left(n\right)=\omega_{1}g(n)+\omega_{2}d(n)+G(n-1)$$

### 3.4. General Flow of the Algorithm

Although the existing mobile robot path planning under rough terrain has been improved with the ground model and the corresponding cost function, the original improved A* algorithm needs to be improved to ensure the effectiveness of the improved A* algorithm because the original improved A* algorithm is not adapted to the existing model. 

Firstly, the node search method of the A* algorithm is improved, and the node search methods used by the existing commonly used A* algorithm include four-neighborhood search, eight-neighborhood search, sixteen-neighborhood search, and twenty-four-neighborhood search. However, due to the unique characteristics of the ground model, the 16-neighborhood search and 24-neighborhood search will pass through the ground where too many nodes are located in the raster, leading to the failure of the ground model. At the same time, the four-neighborhood search generates a smaller range of nodes, and the eight-neighborhood search can balance the number of nodes and the search efficiency to a certain extent. At the same time, it can consider the terrain’s complexity, which makes the path planning more accurate and efficient, so the eight-neighborhood search is adopted as the search method to improve the A* algorithm.

Secondly, the cost of each node in the A* algorithm is calculated as *F*(*n*) = *G*(*n*) + *H*(*n*), where *F*(*n*) is the evaluation cost from the start node to the target node, *G*(*n*) is the evaluation cost from the start node to the current node, and *H*(*n*) is the predicted evaluation cost from the current node to the target node.

Although, according to (9), it can be known to improve the A* algorithm *G*(*n*) calculation method, it also needs *d*(*n*) and *H*(*n*) distance cost design to determine *F*(*n*); the current commonly used distance cost function is mainly used in the Manhattan distance, Euclidean distance, etc. As the Manhattan distance is only applicable to the four-domain search strategy of the node search strategy, the Euclidean distance is used as the distance cost, *d*(*n*), as shown in (10), and *H*(*n*) is shown in (11).

(10) $$d(n)=\sqrt{{\left(x_{n-1}-x_{n}\right)}^{2}+{\left(y_{n-1}-y_{n}\right)}^{2}}$$

(11) $$H(n)=\sqrt{{\left(x_{goal}-x_{n}\right)}^{2}+{\left(y_{goal}-y_{n}\right)}^{2}}$$

In (10) and (11), *x_n_*_−1_ and *y_n−_*_1_ are the x-coordinate and y-coordinate of node *n*−1, *x_n_* and *y_n_* are the *x*-coordinate and *y*-coordinate of node *n*, and *x*_goal_ and *y*_goal_ are the *x*-coordinate and y-coordinate of the target node *n_goal_*, respectively.

Given the above improvement method and the original A* algorithm process, Algorithm 1 shows the overall process of the improved A* algorithm.
**Algorithm 1** The Improved A* Algorithm  1: Initialize OPEN list (to the empty list)  2: Initialize CLOSED list (to the empty list)  3: Create goal node; call it node_goal  4: Create start node; call it node_start  5: Add node_start to the OPEN list  6: **while** the OPEN list is not empty **do**  7:  Get node *n* off the OPEN list with the lowest *F*(*n*)  8:  Add *n* to the CLOSED list  9:  **if** *n* is the same as node_goal **then**10:   we have found the solution; return Solution(*n*)11:  **else:** Generate each successor node *n*’ of *n*12:  **end if**13:  **for** each successor node *n*’ of *n* obtained by an 8-neighborhood search **do**14:   Set the parent of *n*’ to *n*15:   Set *H*(*n*’) to be the heuristically estimate distance to node_goal16:   Set *a*, *h*, *S* to be the ground model values from node *n* to *n*’17:   Set *g*(*n*’)to be the elevation cost from node *n* to *n*’18:   Set *d*(*n*’)to be the distance cost from node n to *n*’19:   Set *G*(*n*’) =*ω*_1_*g*(*n*’)+*ω*_2_*d*(*n*’)+*G*(*n*)20:   Set *F*(*n*’)= *G*(*n*’)+ *H*(*n*’)21:   **if** *n*’ is on the OPEN list and the existing one is as good or better **then**22:     discard *n*’ and continue23:   **end if**24:   **if** *n*’ is on the CLOSED list and the existing one is as good or better **then**25:     discard *n*’ and continue26:   **end if**27:   Remove occurrences of *n*’ from OPEN and CLOSED28:   Add *n*’ to the OPEN list29: **end for**30: **end while**31: **return** failure (if we reach this point, we’ve searched all reachable nodes and still haven’t found the solution, therefore one doesn’t exist)

## 4. Simulation and Analysis

**Figure 4:**
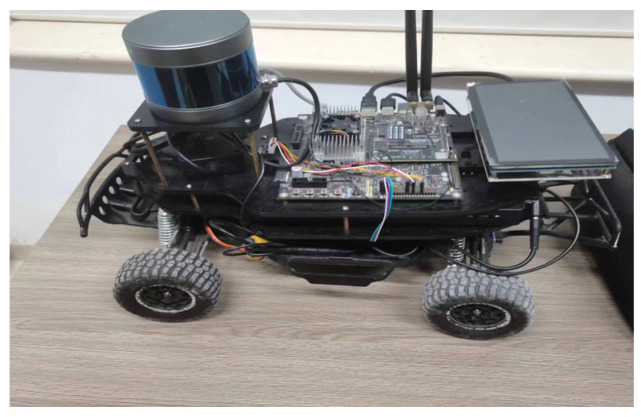
Ackermann steering mobile robot platforms. The robot is built with 3D LiDAR for sensing and is processed and controlled by Nvidia Jetson TX2 running Ubuntu 18.04 and ROS 1.12.17.

The performance improvement in the algorithm under the rough terrain proposed in this paper is verified. Firstly, the simulations are carried out through MATLAB R2022a and ROS 1.12.17, and the algorithm’s effectiveness is verified in improving the ruggedness of the path and the optimality of the path. Since the paths generated by ROS and physical experiments must follow a quadratic plan, this may mask the superiority of the original algorithm. Therefore, a combination of MATLAB R2022a simulation experiments can better reflect the algorithm’s superiority; thus, MATLAB and ROS are used for simulation in this paper. Before the simulation and experiments are carried out, preliminary analysis and design of the experimental equipment and experimental parameters are carried out, so the Ackermann steering mobile robot platform shown in Figure 4 is used to perform all the tests. The robot is built with 3D LiDAR for sensing and is processed and controlled by Nvidia Jetson TX2 running Ubuntu 18.04 and ROS 1.12.17. According to the parameters of the mobile robot design algorithm, parameters are designed, as shown in Table 1.

**Figure 5:**
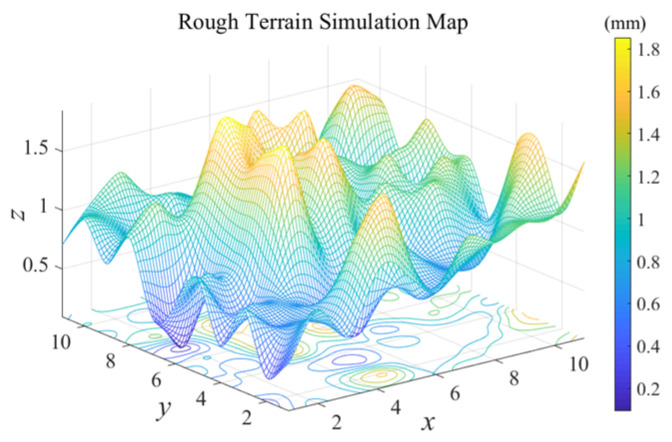
MATLAB rough terrain simulation map.

For rough terrains, the elevation map construction method and interpolation method in MATLAB were used to construct a simulation map of rough terrain, as shown in Figure 5. This map simulates the ground’s rough terrain characteristics, and (0.5, 0.5, 0.5) and (9.5, 9.5, 1) were selected as the starting and goal points.

**Figure 6:**
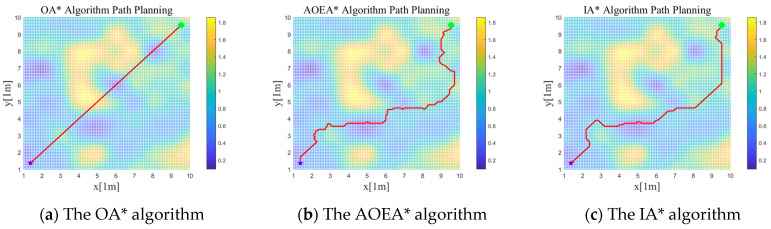
Paths formed by the mobile robot path planning in MATLAB rough terrain simulation map: (**a**) shows the path formed by the OA* algorithm; (**b**) shows the path formed by the AOEA* algorithm; and (**c**) shows the path formed by the IA* algorithm.

Since the simulation needs to verify the superiority of the algorithm, the algorithms used are the improved A* (IA*) algorithm, the A* algorithm with added obstacle expansion (AOEA*), the most common algorithm currently used by mobile robots, and the original A* (OA*) algorithm for verification, which will be verified by four indexes, the total distance of the path *l*_v_, the average slope at path nodes *a*_v_, the average height difference at path nodes *h*_v_, and the average ruggedness of the ground at path node *S*_v_. The final results are obtained as shown in Figure 6. The experimental results of the four parameter values obtained by three path planning methods are shown in Table 2 below. 

According to Figure 6 and Table 2, the *a*_v_, *h*_v_, and *S*_v_ in the paths planned by the IA* algorithm are much lower than those of the OA* algorithm under rugged terrain, thus proving that the IA* algorithm selects relatively smooth ground under rugged terrain. The total length of the paths planned by the IA* algorithm is 20.8% less than the total length of the paths planned by the AOEA* algorithm under rugged terrain, thus proving the optimality of the IA* algorithm. In summary, the IA* algorithm performs better in terms of distance, ground trafficability, and ground ruggedness, which verifies the effectiveness of the IA* algorithm on the data.

Since MATLAB simulation does not reflect the algorithm’s effectiveness in the application of mobile robots, it is necessary to carry out further simulations to verify the performance of the mobile robot after the application of the algorithms. Therefore, the algorithm must be simulated through ROS. Firstly, Gazebo’s model library creates a simulation environment reflecting the characteristics of the rough terrain. Secondly, the mobile robot simulation is performed to model the mobile robot according to the size of the mobile robot used in the experiment, the type of sensors constructed, and their locations. Finally, to illustrate the effectiveness of the IA* algorithm, since the AOEA* algorithm is higher than the OA* algorithm in terms of application efficiency, the IA* algorithm and the AOEA* algorithm are used for comparative validation and evaluated in terms of the total length of the paths and the angle between the coordinate axes of the mobile robot coordinate system and the coordinate axes of the ground coordinate system.

**Figure 7:**
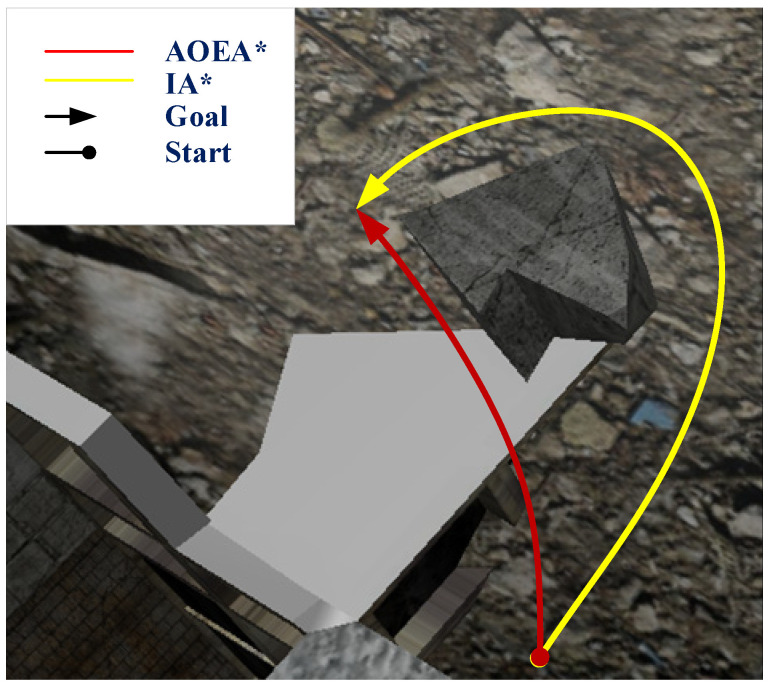
Paths are formed by the mobile robot path planning in the Gazebo rough terrain simulation, in which the red path is the path created by the AOEA* algorithm, and the yellow path is the path made by the IA* algorithm. Arrows and dots represent the start and end points of paths.

**Figure 8:**
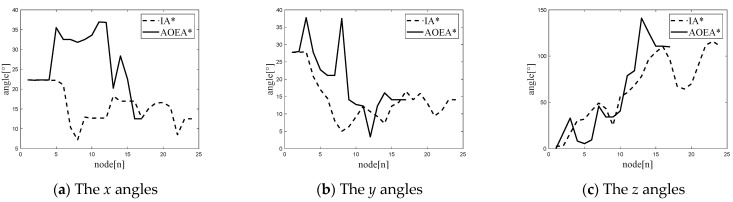
The *x*-, *y*-, and *z*-axis angles of each node of the mobile robot to the ground coordinate system for the paths planned by the two algorithms in the Gazebo rough terrain simulation: (**a**) shows the *x* angles of each node of the mobile robot to the ground coordinate system for the paths planned by the two algorithms in the Gazebo rough terrain simulation; (**b**) shows the *y* angles of each node of the mobile robot to the ground coordinate system for the paths planned by the two algorithms in the Gazebo rough terrain simulation; and (**c**) shows the *z* angles of each node of the mobile robot to the ground coordinate system for the paths planned by the two algorithms in the Gazebo rough terrain simulation.

The ROS system simulates the IA* and AOEA* algorithms, and the paths shown in Figure 7 are finally obtained. The AOEA* algorithm forms the red path, and the yellow path is created by the IA* algorithm. The angle between the node mobile robot and the ground plane coordinate system in the paths formed by the two algorithms is compared, and the angle comparison diagram shown in Figure 8 is obtained. The paths planned by the IA* algorithm and the AOEA* algorithm will be analyzed by the following seven parameters: the total path distance *l*_v_, the average value of the angle between the x-axis of the mobile robot coordinate system and the *x*-axis of the ground coordinate system at each node *θ_x_*, the average value of the angle between the *y*-axis of the mobile robot coordinate system and the y-axis of the ground coordinate system at each node *θ_y_*, the average value of the angle between the *z*-axis of the mobile robot coordinate system and the z-axis of the ground coordinate system at each node *θ_z_*, the maximum value of the angle between the x-axis of the mobile robot coordinate system and the x-axis of the ground coordinate system at each node *θ_x_*_max_, the maximum value of the angle between the *y*-axis of the mobile robot coordinate system and the y-axis of the ground coordinate system at each node *θ_y_*_max_, and the maximum value of the angle between the *z*-axis of the mobile robot coordinate system and the *z*-axis of the ground coordinate system at each node *θ_z_*_max_. The average angle and maximum angle value of the path nodes along the three coordinate axes of the two path planning methods are shown in Table 3.

Table 3 shows that under rough terrain, the average and maximum angles between the mobile robot coordinate system and the nodes of the ground plane coordinate system in the path planned by the IA* algorithm are reduced by 34.41% and 34.97%, respectively, compared with those of the path intended by the AOEA* algorithm. The path planned by the IA* algorithm is 11.437 m, and the path planned by the AOEA* algorithm is 9.581 m. The IA* algorithm increases by only 20.01% compared with the AOEA* algorithm, which proves that the IA* algorithm improves the degree of smoothness of the path under the condition of ensuring that the length of the path does not change substantially, thus verifying the optimality of the IA* algorithm.

## 5. Real-Life Experiment and Analysis

**Figure 9:**
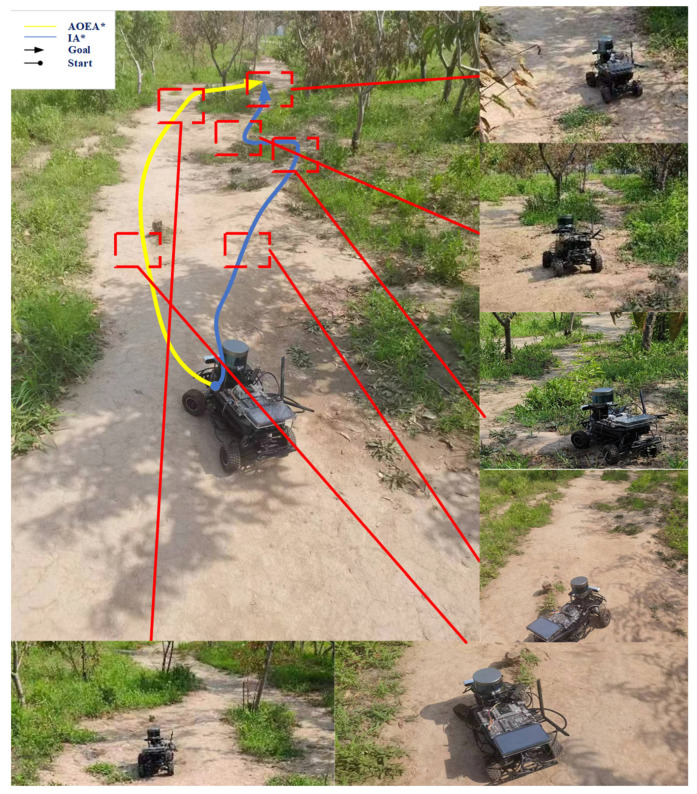
Paths are formed by the mobile robot path planning in the real-life environment, in which the yellow path is the path created by the AOEA* algorithm, and the blue path is the path made by the IA* algorithm. Arrows and dots represent the start and end points of paths.

**Figure 10:**
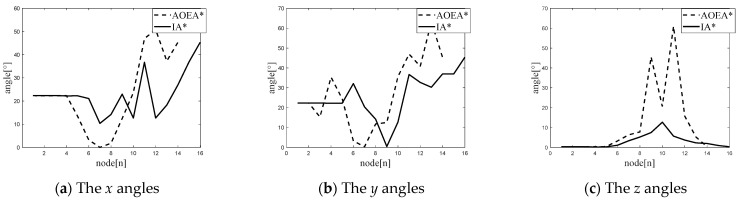
The *x*-, *y*-, and *z*-axis angles of each node of the mobile robot to the ground coordinate system for the paths planned by the two algorithms in the Gazebo rough terrain simulation: (**a**) shows the *x* angles of each node of the mobile robot to the ground coordinate system for the paths planned by the two algorithms in the Gazebo rough terrain simulation; (**b**) shows the *y* angles of each node of the mobile robot to the ground coordinate system for the paths planned by the two algorithms in the Gazebo rough terrain simulation; and (**c**) shows the *z* angles of each node of the mobile robot to the ground coordinate system for the paths planned by the two algorithms in the Gazebo rough terrain simulation.

Since simulation using only MATLAB and ROS can only verify the superiority of the algorithm in the simulation, we also need to prove it through a real-life experiment. Since the experimental environment requires the mobile robot to navigate under the AOEA* algorithm with the problem of center-of-mass instability, we designed an environment in which the mobile robot through the AOEA* algorithm occurs with the instability of the center of mass of the mobile robot. Afterward, the IA* algorithm and AOEA* algorithm are experimented with in this environment. The obtained paths are shown in Figure 9, where the yellow path is the path formed by the path planning of the AOEA* algorithm, and the blue path is the path created by the path planning of the IA* algorithm, and the angle comparison diagram shown in Figure 10 is obtained.

The paths planned by the IA* algorithm and the AOEA* algorithm will be analyzed by the following seven parameters: the total path distance *l*_v_, the average value of the angle between the *x*-axis of the mobile robot coordinate system and the *x*-axis of the ground coordinate system at each node *θ_x_*, the average value of the angle between the *y*-axis of the mobile robot coordinate system and the *y*-axis of the ground coordinate system at each node *θ_y_*, the average value of the angle between the *z*-axis of the mobile robot coordinate system and the *z*-axis of the ground coordinate system at each node *θ_z_*, the maximum value of the angle between the *x*-axis of the mobile robot coordinate system and the *x*-axis of the ground coordinate system at each node *θ_x_*_max_, the maximum value of the angle between the *y*-axis of the mobile robot coordinate system and the *y*-axis of the ground coordinate system at each node *θ_y_*_max_, and the maximum value of the angle between the *z*-axis of the mobile robot coordinate system and the *z*-axis of the ground coordinate system at each node *θ_z_*_max_. The path values are shown in Table 4.

Table 4 shows that under rough terrain, the average and maximum angles between the mobile robot coordinate system and the nodes of the ground plane coordinate system in the path planned by the IA* algorithm are reduced by 45.15% and 38.59%, respectively, compared with those of the path planned by the AOEA* algorithm. The path planned by the IA* algorithm is 12.732 m, and the path planned by the AOEA* algorithm is 10.936 m. The IA* algorithm increases by only 14.11% compared with the AOEA* algorithm, which proves that the IA* algorithm improves the degree of smoothness of the path under the condition of ensuring that the length of the path does not change substantially, thus verifying the optimality of the IA* algorithm.

## 6. Discussion

The traditional path planning for mobile robots and the improved path planning for mobile robots under rough terrain have the problem of center-of-mass instability when applied to mobile robots, and the center-of-mass instability of wheeled robots under rough terrain after using the above path planning algorithms is mainly caused by two problems. Firstly, although the above algorithm models the environment of rough terrain and constructs a ground model, it only considers whether the ground is passable or not after creating the ground model but does not consider whether the ground has smooth trafficability or not, i.e., the constructed ground does not consider the roughness of the ground. Secondly, the cost design of the above algorithms for the ground model is designed using methods such as classification and priority comparison, which leads to the need for more optimality of the algorithms and the existence of the generated paths with a significant degree of ruggedness. Therefore, this study aims to propose an IA* algorithm to solve the above problems and avoid the center-of-mass instability of mobile robots under rough terrain.

Subsequent simulation experiments and physical experiments of the IA* algorithm show that the proposed algorithm significantly improves the smoothness of the mobile robot under rough terrain and avoids the center-of-mass instability of the mobile robot compared to the traditional and improved algorithms. The experimental results show that the IA* algorithm generates reasonable paths under rough terrain. Compared with the AOEA* algorithm, the path generated by the IA* algorithm can reduce the path’s ruggedness, improve the wheeled robot’s smoothness, and increase the length of the path by only 15–20% relative to the original path. The IA* algorithm proposed in this study is of great practical significance for the movement of mobile robots under rough terrain, and it can help mobile robots plan the optimal path safely and avoid center-of-mass instability. Although the IA* algorithm performs well under rough terrain, there are still some limitations, e.g., the current ground transportation stability model only considers the two models of slope and height difference, which still cannot reflect the specific degree of ground undulation. There is still a certain degree of error. In the future, ground features can be further designed to improve the reflection of the degree of ground undulation.

## 7. Conclusions

In this paper, an improved A* algorithm for mobile robots in rugged terrain is designed based on the ground trafficability model and the ground ruggedness model based on elevation maps by considering the center-of-mass instability problem of mobile robots in rough terrain. Firstly, the ground trafficability and ruggedness models are designed for the characteristics of rugged terrain and elevation maps, which can reflect the search path’s ground trafficability and ground smoothness. Secondly, according to the ground trafficability model and the ground ruggedness model, a continuous cost function is designed to match the characteristics of rugged terrain with the two types of models to improve the optimality of the algorithm and avoid the problem of an unstable center of mass. Finally, the smoothness and path optimality of the algorithm are verified by simulation and experiment, proving the algorithm’s effectiveness in rugged terrain. Future research is directed towards further designing the ground model to improve the degree of responsiveness to ground attributes and to apply it to the broader field of autonomous navigation for mobile robots under rough terrain.

## Figures and Tables

**Table 1:** Values of each parameter of the algorithm.

Parameter	Value
*h_y_*/m	0.05
*a_y_*/rad	0.5
*S_y_*/m^2^	0.02
*ω*_p_	0.33
*ω*_e_	0.33
*ω*_q_	0.34
*ω*_1_	0.5
*ω*_2_	0.5

**Table 2:** Experimental results of four parameter values obtained by three path planning methods.

Algorithm	The Total Distance of the Path (*l*_v_/m)	The Average Slope at Path Nodes (*a*_v_/rad)	The Average Height Difference at Path Nodes (*h*_v_/m)	The Average Ruggedness of the Ground at Path Nodes (*S*_v_/m^2^)
OA*	14.38	0.58	0.097	0.049
AOEA*	19.32	0.31	0.034	0.014
IA*	17.54	0.26	0.027	0.013

**Table 3:** The average angle and maximum angle value of the path nodes along the three coordinate axes of the two path planning methods.

Algorithm	*l*_v_/m	*θ_x_*/°	*θ_y_*/°	*θ_z_*/°	*θ_x_*_max_/°	*θ_y_*_max_/°	*θ_z_*_max_/°
IA*	11.437	2.01	2.40	10.84	12.45	9.84	50.34
AOEA*	9.851	3.76	2.92	17.65	17.34	24.17	60.27

**Table 4:** Path parameters formed by mobile robot path planning under rough terrain in a real-life environment.

Algorithm	*l*_v_/m	*θ_x_*/°	*θ_y_*/°	*θ_z_*/°	*θ_x_*_max_/°	*θ_y_*_max_/°	*θ_z_*_max_/°
IA*	12.732	5.87	5.94	1.88	18.68	24.03	6.98
AOEA*	10.936	5.97	11.71	12.14	23.33	27.09	45.12

## Data Availability

The original contributions presented in the study are included in the article, further inquiries can be directed to the corresponding author.
